# Mapping diversity in gender identity and gender roles across sex and age in the Dutch general population: a large-scale cohort study

**DOI:** 10.1016/j.eclinm.2025.103359

**Published:** 2025-07-28

**Authors:** Sarah M. Burke, Daniëlle B.A. Kroeze, S. Lucette Kiewiet, Aranka V. Ballering

**Affiliations:** a Department of Psychiatry, University of Groningen, University Medical Centre Groningen, P.O. Box 30.001, 9700 RB, Groningen, the Netherlands; b Department of Plastic Surgery, University of Groningen, University Medical Centre Groningen, P.O. Box 30.001, 9700 RB, Groningen, the Netherlands; c Faculty of Political and Social Sciences, Department of Sociology, Ghent University, Sint-Pietersnieuwstraat 41, 9000, Ghent, Belgium

**Keywords:** Gender diversity, General population, Femininity, Masculinity, Multidimensional

## Abstract

**Background:**

Knowledge of diversity in gender identity and gender roles in the general population is limited. This study aimed to report the prevalence estimates of gender identity and gender roles among the adult general population, stratified by age and sex.

**Methods:**

In the third general assessment of the prospective Dutch Lifelines Cohort Study, conducted between 2019 and 2023, sex and current gender identity were assessed using a self-reported categorical item, in which participants aged 18 years and older could indicate their sex assigned at birth (male or female) and current gender identity (man or woman), or select the option with a free-text field. Two separate dimensional measures assessed adherence to feminine and masculine gender roles. Using a cross-sectional study design, we describe the distribution of gender identities and adherence to gender roles, stratified by age and sex as registered by the municipality. Differences herein are assessed via independent t-tests and ANOVA.

**Findings:**

A total of 63,190 participants (mean age = 55.4 years [SD = 12.6]) were included in the study. Most participants identified as cisgender (36,835 [58.6%; 95% CI = 58.2–58.9] cisgender women; 25,893 [41.2%; 95% CI = 40.8–41.6] cisgender men). 66 (0.11%; 95% CI = 0.08%–0.13%) participants identified as non-cisgender. Among cisgender participants registered as males, masculine gender role scores increased across age groups, with younger individuals (18–30 years) scoring lower (M = 9.3, SD = 1.2) than older individuals (71–97 years; M = 9.7, SD = 1.0; *F*_(5,25925)_ = 35.5; *p* < 0.0001; η^2^ = 0.008 [95% CI = 0.006–0.010]). A similar pattern was observed for adherence to feminine gender roles among cisgender participants registered as females, where younger individuals (M = 9.1, SD = 1.2) scored lower than older individuals (M = 9.7, SD = 1.0; *F*_(5,36900)_ = 137.2; *p* < 0.0001, η^2^ = 0.018 [95% CI = 0.016–0.021]). Cisgender participants registered as male reported stronger adherence to masculine roles (M = 9.6, SD = 1.0), than their female counterparts to feminine roles (M = 9.3, SD = 1.2; t_(60,459)=_27.7; *p* < 0.0001, Cohen's *d* = 0.218 [95% CI = 0.202–0.234]).

**Interpretation:**

Although effect sizes are small, younger and female individuals indicate greater diversity in gender roles than older and male individuals, respectively. This shows that diversity in gender role adherence is common. A limitation of this study is the relatively older sample, which limits representation of younger individuals and may affect generalizability. These findings have implications for clinical practice and policy, as recognizing gender role diversity could help healthcare providers tailor interventions and assessments. Given the small but meaningful effect sizes, continued research on gender roles and their health impact across age groups is warranted to inform gender-sensitive policies.

**Funding:**

The 10.13039/501100002999Dutch Ministry of Health, Welfare and Sport, the Dutch Ministry of Economic Affairs, the 10.13039/501100005075University Medical Center Groningen (UMCG), Groningen University, the Provinces in the North of the Netherlands (Drenthe, Friesland, Groningen) and 10.13039/501100001826ZonMw (The Netherlands Organization for Health Research and Development).

**Research in context**

**Evidence before this study**

We searched PubMed up to Jan 01, 2025 for studies published in Dutch or English that investigated gender diversity among the general population. We searched for studies that explored gender identity and gender in terms of adherence to feminine and masculine gender roles in large-scale general population cohort studies. We did not perform a formal systematic review. National surveys and census studies conducted in North America and Europe have reported estimates regarding diversity in gender identity ranging from 0.1% to 2.3%. This variety is due to studies applying different methods, measures and definitions for gender diversity. The majority of previous research assessing these topics focused on adolescents, were performed in clinical settings prone to selection bias, used small study samples, or limited themselves to gender identity, while disregarding the implications of gender role adherence for health. Therefore, reliable, robust and adequate estimates regarding diversity in gender identity and gender roles among the general population are scarce. Our study addressed these issues.

**Added value of this study**

To our knowledge, this study is one of the first to investigate gender diversity, operationalised by a categorical measure of gender identity and dimensional measures of adherence to masculine and feminine gender roles, across sex registered by the municipality and age, in the general population. The use of Lifelines data enabled us to examine this in a large dataset (N = 63,190) with minimal risk of selection bias.

**Implications of all the available evidence**

Our approach allows us to present the patterns of gender identity and levels of adherence to masculine and feminine gender roles across age and sex registered by the municipality. We find that the vast majority of participants considers themselves as cisgender, with 0.11% (95% CI = 0.08%–0.13%) of the participants explicitly indicating a non-cisgender identity. The variation of gender roles, among cisgender participants, over sex and age is substantial. Overall, we find that younger participants and female participants experience more diversity in adherence to gender roles than older and male participants. Most importantly, we show that diversity in gender identity and roles is inherent to the general population, and cannot, nor should, be disregarded, especially in health-related research.

## Introduction

Sex and gender-related factors are known to strongly influence health.^1^ Their influences are found across virtually all medical disciplines and all aspects of patients’ illness trajectories.^2^, ^3^, ^4^ For example, female sex, as well as adherence to feminine gender roles have been found to associate with the prevalence and persistence of common somatic symptoms in the general population, as well as with primary care help-seeking behavior for these symptoms (Box 1).^10^, ^11^, ^12^, ^13^ Adherence to feminine gender roles, often measured via compliance with roles traditionally ascribed to women, such as household responsibilities, caregiving responsibilities, and reduced paid working hours, have been shown to associate with decreased access to timely and adequate healthcare in both men and women.^14^ Furthermore, adherence to masculine gender roles is thought to be protective against somatic and psychological symptoms.^13^^,^^15^ This is potentially mediated by an increase in agency, which is strongly tied to masculine gender. Despite the substantial effects of sex, gender, and diversity herein on health outcomes, there is a lack of knowledge on the exact mechanisms of these effects. Moreover, there are fundamental knowledge gaps regarding the prevalence of diversity regarding gender identity and gender roles in the general population. This hinders quality of care and progression towards personalised healthcare.

Box 1 **Glossary of terms related to sex, gender, and diversity herein**

**Table:** 

Sex	Refers to the biological characteristics, among other genes, hormones, physiology, and anatomy, of female, male and intersex bodies. Sex is assigned at birth. In cohort studies, multiple approaches to measure sex, including genetics, self-reported sex at birth, or sex derived from registries are available. Although sex is frequently considered as dichotomy (i.e., male *versus* female), sex characteristics exist on a continuum, challenging binary beliefs about biological sex.
Gender identity	Gender identity refers to a person's deeply felt internal sense of gender, whether man, woman, or beyond that, such as non-binary or agender. An individual's gender identity may or may not be the same as their sex registered at birth.
Cisgender	Refers to individuals whose gender identity aligns with the sex they were assigned at birth. For example, a person assigned female at birth who identifies as a woman is considered cisgender. In the context of this study, we use non-cisgender (identity) if an individual's gender identity does not align with the sex they were assigned at birth.
Transgender or gender diverse	Refers to individuals whose gender identity differs from the sex they were assigned at birth. This term is often used as an umbrella category that includes a variety of gender identities.
Transgender Men (Trans Men)	Refers to individuals who were assigned female at birth, but identify as men. Individuals may undergo medical treatments, such as hormone therapy or surgery to align their physical appearance with their gender identity, but this is not a requirement for being recognized as a trans man.
Transgender Women (Trans Women)	Refers to individuals who were assigned male at birth, but identify as women. Similar to trans men, they may pursue medical treatments to align their physical appearance with their gender identity, though this is not necessary for their identity as trans women.
Non-binary Gender Identity	Refers to a gender identity that does not conform to the traditional binary classification of male or female (including trans men and trans women). Individuals who identify as non-binary may experience their gender as a blend of both, neither, or as an entirely separate category.
Gender Role	Refers to the societal expectations and norms associated with a particular gender. These roles are imposed upon individuals by society, and dictate how individuals should behave, dress, and interact based on their perceived gender. What is considered a traditional gender role can vary significantly across cultures and time periods.

Note: definitions are based on prior studies; Ballering et al., Arch Sex Behav, 2023,^5^ Office for National Statistics England and Wales, 2021,^6^ Kennis, 2024,^7^ Polderman et al., Behav Gen, 2018,^8^ Coleman et al., Int J Trans Health, 2022.^9^

Multiple reasons for these knowledge gaps have been described. First, despite their intertwinement, sex and gender are different concepts. Nevertheless, they are frequently conflated in research.^16^ Sex refers to biological characteristics of female, male, and intersex bodies (e.g., anatomy, hormonal levels, and genetics). In contrast, gender comprises a multidimensional and socioculturally-constructed concept that includes the embodiment of identities, roles, behaviours, relations and hierarchies by women, men, and non-binary individuals as prescribed by social norms.^5^ The conflation of sex and gender blurs their relative contributions to health and disease, subsequently affecting the rigor and validity of research results. Second, sex and gender-sensitivity were historically mainly considered relevant to women's reproductive health.^17^ This history still impacts modern medicine. For example (bio)medical research beyond reproductive medicine during the second half of the twentieth century included mainly male participants, rendering researchers with little information about the female body.^18^ Third, researchers may presume that sex and gender are controversial topics, are too difficult to interpret, or are too sensitive to answer for participants.^19^ As a result, it is assumed that assessing these topics in cohort studies impacts participant retention. Such assumptions are problematic, as they result in omitting the assessment of sex and gender, which reinforces and reifies an unjustified irrelevance hereof.

The relevance of adequately assessing sex and gender to obtain information on the sexed and gendered mechanisms that affect health has been acknowledged in clinical cohorts, predominantly in the context of adolescence.^20^ In a recent Dutch longitudinal study among 2772 participants, 2.4% of the 11-year-olds indicated to often wish to be of the opposite sex, with this proportion decreasing over time to 0.8% of 26-year-olds. Although large-scale general population cohort studies have been lagging behind in this aspect, an increasing number of measures that assess sex and gender have recently been introduced.^19^, ^20^, ^21^ Participants' sex can be, for instance, assessed via self-reported measures,^19^ derived from municipal registries,^13^ or based on genetic information.^22^ To assess aspects of participants' gender, multiple self-report measures are becoming available. In addition, various large-scale cohort studies have introduced composite gender scores, which describe the compliance of participants with roles and characteristics traditionally ascribed to women and men.^23^, ^24^, ^25^, ^26^ However, these secondary measures are based on previously collected data and cannot capture one's gender identity.

To incorporate and acknowledge the lived experiences of people and to obtain more robust and valid data on gender and diversity herein, self-reported gender measures are increasingly introduced in large-scale data-collection efforts. For example, the US Veterans Health Administration has recently introduced a measure on self-reported gender identity reporting that 0.11% of 66,348 respondents identifies as transgender or gender diverse.^27^ The CDC's Behavior Risk Factor Surveillance System also provided respondents with the option to indicate a transgender identity, reporting that among the US general population, an estimated 1.3% of the 18-to-24-year-olds identifies as transgender, which decreases to 0.3% of individuals aged over 65.^6^ Statistics Canada reports in their 2022 census release that 59,460 (0.2%) and 41,350 (0.1%) respondents indicated a transgender or non-binary identity, respectively,^7^ and in the 2021 census in England and Wales 262,138 (0.6%) of the respondents indicated that they did not identify with their sex registered at birth.^28^ Statistics Netherlands used a sample of 182,218 individuals to estimate that 0.4%, 0.3%, and 0.3% of the Dutch general population identifies as transgender man, transgender woman, or as non-binary, respectively.^29^ The variety in these estimates could be due to cultural differences related to gender, but also to different gender measures applied. Self-reported levels of feminine and/or masculine gender roles were often not included. These are assessed in smaller study samples. For example, a US-based cross-sectional study among 1514 adult cisgender respondents reports that 72% and 68% of male and female participants did not consider themselves fully masculine or fully feminine, respectively.^30^ These proportions decreased with age.^30^ This indicates variance in gender roles among the general population, which aligns with research exploring gender identity-related experiences.^31^

Also within the large-scale Dutch general population cohort Lifelines a self-reported gender measure was introduced.^19^ This measure combines a categorical item inclusively assessing self-reported sex assigned at birth and current gender identity, with two separate dimensional items that assess adherence to feminine and masculine gender roles in both men and women (Appendix 1). These data allow to distinguish between cisgender and non-cisgender populations and to separately describe the variations in gender role patterns.

We used the data from the Lifelines Cohort study, to provide an overview of the prevalence estimates of gender identities and gender roles and diversity herein among the general population. We aimed to quantify participants’ (1) self-reported gender identities, including sex assigned at birth and their (2) self-reported adherence to feminine and masculine gender roles, stratified by age and sex as registered by the municipality.

## Methods

### Participants

Lifelines is a multi-disciplinary prospective population-based cohort study examining in a unique three-generation design the health and health-related behaviours of 167,729 persons living in the North of The Netherlands. It employs a broad range of investigative procedures in assessing the biomedical, socio-demographic, behavioural, physical and psychological factors which contribute to the health and disease of the general population, with a special focus on multi-morbidity and complex genetics. Extensive information on the cohort, including its representativeness and methods of recruitment is provided elsewhere.^32^ The self-reported gender measure was introduced during Lifelines’ third regular assessment round, which ran from 2019 to 2024.^19^ The Lifelines Cohort Study and its add-on studies were approved by the Medical Ethical Committee of University Medical Centre Groningen (2007/152) and participants provided written informed consent to take part. In writing this report, we adhered to the STROBE guidelines.^33^

### Assessment of sex and gender

In Lifelines, participants' sex was derived from the municipal registry,^13^ which for most participants corresponds to sex assigned at birth. However, the Dutch law allows for individuals to change their sex registration in the municipal registry following a written request to change one's gender registration to the Registrar of Civil Status, and, depending on the individual's age this may require approval of a petition by the court.

Assessment of gender identity consisted of a categorical item that assesses both self-reported sex assigned at birth and current gender identity. Participants could select one of four binary options (male or female sex assigned at birth in combination with cisgender or transgender identity) or select a fifth option with a free-text field if participants did not consider any of the provided categorised answer options applicable to them (Appendix 1).

A second item assessing gender roles consisted of dimensional measures that allowed for participants to indicate the degree to which they considered themselves adhering to masculine gender roles and, on a separate scale, feminine gender roles with possible responses from “1—Strongly disagree” to “10—Strongly agree”. The statements were preceded by a short introduction explaining that both men and women may consider themselves masculine or feminine, for example based on characteristics or hobbies that most people typically associate with masculinity or femininity, and that some people may consider themselves neither masculine nor feminine. Thereafter, masculinity and femininity were assessed on separate continua, as we do not consider femininity to oppose masculinity, but rather as two separate dimensions that may overlap within individuals (Appendix 1).^34^^,^^35^ The gender measure was not formally validated and developed specifically for the Lifelines Cohort Study. It was developed in close collaboration with a gender diverse participant panel to ensure optimal face and content validity.^19^

### Analyses

Participants were divided into six age groups, namely 18–30, 31–40, 41–50, 51–60, 61–70, and 71+ years. Prevalences of answers to the categorical gender item, assessing self-reported sex assigned at birth and gender identity, are provided as absolute and relative frequencies, stratified by sex as registered by the municipality and age group.

The free-text answers were analysed by two independent coders (SK, DK) and subsequently thematically grouped via consensus discussion into categories that were inductively defined. Prevalences of the categories derived from the free-text field responses are provided as absolute and relative frequencies.

Prevalences of self-reported adherence to masculine and feminine gender roles are provided as absolute and relative frequencies, stratified by sex as registered by the municipality and age group. We defined three subgroups in terms of adherence to gender roles, but these were not formally validated. First, we defined a group of “specifically highly gendered” participants who considered themselves either predominantly masculine *or* feminine, but scored low on the respective other gender dimension (i.e., scoring ≥8 on masculinity and ≤2 on femininity and vice versa). Second, we defined those who considered themselves masculine *and* feminine as “non-specifically highly gendered” (i.e., scoring ≥5 on masculinity and femininity), and third, participants who considered themselves *neither* strongly masculine *nor* feminine as “not highly gendered” (i.e., scoring <5 on masculinity and femininity).

One-way ANOVA, complemented by a Bonferroni post hoc test, was applied to assess whether mean masculinity and femininity scores differed between age groups. We provide eta squared (η^2^) as an indication of effect size (proportion of explained variance), with η^2^ ≈ 0.01 indicating a small effect, η^2^ ≈ 0.06 indicating a medium effect, and η^2^ ≈ 0.14 or higher indicating a large effect. Finally, using independent t-tests, we tested whether participants who were municipally registered as male scored differently on the masculinity measure than participants who were municipally registered as females scored on the femininity measure. We provide Cohen's d as an indication for the effect size between the two means, in which 0.2 ≤ d ≤ 0.5 indicates a small effect, 0.5 ≤ d ≤ 0.8 a medium effect, and d ≥ 0.8 a large effect. Individuals with missing values on the categorical gender identity item, the two-dimensional masculinity and femininity measures, age, or sex as registered by the municipality were excluded from analyses.

### Role of the funding source

The funder of the study had no role in study design, data collection, data analysis, data interpretation, or writing of the report. SB and AB had full access to the data underlying the study and all authors had final responsibility for the decision to submit for publication.

## Results

A total of 63,190 participants [58.7% municipally registered as female, mean age 55.4 years (SD = 12.6; min–max = 18–97)] completed the self-report gender measurement in Lifelines (Table 1).

**Table 1:** Absolute and relative frequencies of gender identities among participants, stratified by municipally registered sex and age group.

Participants municipally registered as male (N = 26,085)^a^							
		Cis woman^b^	Cis man	Trans woman	Trans man^c^	Indicated via free-text^d^	Missing
Total, excluding missing	N = 25,945	6 (0.0%; 0.0–0.0)	25,889 (99.8%; 99.7–99.8)	10 (0.0%; 0.0–0.1)	9 (0.0%; 0.0–0.1)	31 (0.1%; 0.0–0.2)	140 (0.5%)
Age in years							
18–30	N = 833	0 (0%)	828 (99.4%; 98.6–99.8)	1 (0.1%; 0.0–0.7)	2 (0.2%; 0.0–0.9)	2 (0.2%; 0.0–0.9)	2 (0.2%)
31–40	N = 2176	0 (0%)	2172 (99.8%; 99.5–99.9)	0 (0%)	1 (0.0%; 0.0–0.3)	3 (0.1%; 0.0–0.4)	5 (0.2%)
41–50	N = 4489	1 (0%; 0.0–0.1)	4473 (99.6%; 99.4–99.8)	3 (0.1%; 0.0–0.2)	2 (0.0%; 0.0–0.2)	10 (0.2%; 0.1–0.4)	20 (0.4%)
51–60	N = 9054	0 (0%)	9035 (99.8%; 99.7–99.9)	3 (0.0%; 0.0–0.1)	3 (0.0%; 0.0–0.1)	13 (0.1%; 0.1–0.2)	39 (0.4%)
61–70	N = 5735	3 (0.1%; 0.0–0.2)	5728 (99.9%; 99.7–100.0)	2 (0.0%; 0.0–0.1)	1 (0.0%; 0.0–0.1)	1 (0.0%; 0.0–0.2)	36 (0.6%)
71+	N = 3658	2 (0.1%; 0.0–0.2)	3653 (99.9%; 99.7–100.0)	1 (0.0%; 0.0–0.2)	0 (0%)	2 (0.1%; 0.0–0.2)	38 (1.0%)

Participants municipally registered as female (N = 37,105)^a^							
		Cis woman	Cis man^b^	Trans woman^c^	Trans man	Indicated via free-text^e^	Missing
Total, excluding missing	N = 36,894	36,829 (99.8%; 99.8–99.9)	4 (0.0%; 0.0–0.0)	9 (0.0%; 0.0–0.0)	2 (0.0%; 0.0–0.0)	50 (0.1%; 0.1–0.2)	211 (0.6%)
Age in years							
18–30	N = 1701	1691 (99.4%; 98.9–99.7)	0 (0%)	0 (0%)	0 (0%)	10 (0.6%; 0.3–1.1)	7 (0.4%)
31–40	N = 3237	3228 (99.7%; 99.5–99.9)	1 (0%; 0.0–0.2)	0 (0%)	0 (0%)	8 (0.2%; 0.1–0.5)	7 (0.2%)
41–50	N = 6847	6835 (99.8%; 99.7–99.9)	0 (0%)	2 (0.0%; 0.0–0.1)	1 (0.0%; 0.0–0.1)	9 (0.1%; 0.1–0.2)	25 (0.3%)
51–60	N = 13,792	13,771 (99.8%; 99.8–99.9)	2 (0%; 0.0–0.1)	5 (0.0%; 0.0–0.1)	0 (0%)	14 (0.1%; 0.1–0.2)	68 (0.5%)
61–70	N = 7760	7754 (99.9%; 99.8–100.0)	0 (0%)	1 (0.0%; 0.0–0.1)	0 (0%)	5 (0.1%; 0.0–0.2)	49 (0.6%)
71+	N = 3557	3550 (99.8%; 99.6–99.9)	1 (0%; 0.0–0.2)	1 (0.0%; 0.0–0.2)	1 (0.0%; 0.0–0.2)	4 (0.1%; 0.0–0.3)	55 (1.5%)

Total population of participants (N = 63,190)^a^							
		Cis woman	Cis man	Trans woman	Trans man	Indicated via free-text^f^	Missing
Total, excluding missing	N = 62,839	36,835 (58.6%; 58.2–58.9)	25,893 (41.2%; 40.8–41.6)	19 (0.0%; 0.0–0.0)	11 (0.0%; 0.0–0.0)	81 (0.1%; 0.1–0.2)	351 (0.6%)
Age in years							
18–30	N = 2534	1691 (66.7%; 64.9–68.6)	828 (32.7%; 30.9–34.5)	1 (0.0%; 0.0–0.2)	2 (0.1%; 0.0–0.3)	12 (0.5%; 0.2–0.8)	9 (0.3%)
31–40	N = 5413	3228 (59.6%; 58.3–60.9)	2173 (40.1%; 38.8–41.5)	0 (0%)	1 (0.0%; 0.0–0.1)	11 (0.2%; 0.1–0.4)	12 (0.2%)
41–50	N = 11,336	6836 (60.3%; 59.6–60.9)	4473 (39.5%; 38.6–40.4)	5 (0.0%; 0.0–0.1)	3 (0.0%; 0.0–0.1)	19 (0.2%; 0.1–0.3)	45 (0.4%)
51–60	N = 22,846	13,771 (60.3%; 59.6–60.9)	9037 (39.6%; 38.9–40.2)	8 (0.0%; 0.0–0.1)	3 (0.0%; 0.0–0.0)	27 (0.1%; 0.1–0.2)	107 (0.5%)
61–70	N = 13,495	7757 (57.5%; 56.6–58.3)	5728 (42.4%; 41.6–43.3)	3 (0.0%; 0.0–0.1)	1 (0.0%; 0.0–0.1)	6 (0.0%; 0.0–0.1)	85 (0.6%)
71+	N = 7215	3552 (49.2%; 48.1–50.4)	3654 (50.6%; 49.5–51.8)	2 (0.0%; 0.0–0.1)	1 (0.0%; 0.0–0.1)	6 (0.1%; 0.0–0.2)	93 (1.3%)

a Relative frequencies and concomitant 95% CI are based on participants providing a valid answer, thus excluding participants who did not complete the item.

b Participants who were registered by the municipality as male or female, indicated to be assigned female or male sex at birth, respectively. These indications may be due to a misunderstanding of the question (e.g., sex assigned at birth is considered by these participants as current sex, or felt sex), due to a delay in completing the Lifelines survey compared to a potential change in the municipal database related to gender incongruence, or simply be erroneous. We cannot differentiate between these reasons.

c In the Netherlands, individuals who experience gender incongruence may change their record of sex to what best reflects their gender identity. Therefore, participants who are registered by the municipality as male or female, may self-report to be a trans man or trans woman, respectively.

d Evaluation of the free-text answers provided by participants registered as male by the municipality, resulted in a proportion of these participants being categorised as a cis woman (N = 0), cis man (N = 5), trans woman (N = 2), trans man (N = 0). In the table, we provide the answers as indicated by the participants.

e Evaluation of the free-text answers provided by participants registered as female by the municipality, resulted in a proportion of these participants being categorised as a cis woman (N = 13), cis man (N = 0), trans woman (N = 0), trans man (N = 0). In the table, we provide the answers as indicated by the participants.

f Evaluation of the free-text answers provided by participants, resulted in a proportion of these participants being categorised as a cis woman (N = 36,848), cis man (N = 25,898), trans woman (N = 21), and trans man (N = 11). In the table, we provide the answers as indicated by the participants.

### Sex assigned at birth and current gender identity

The majority of participants identified as cisgender, with 36,835 participants (58.3%; 95% CI = 58.2%–59.0%) identifying as cisgender woman and 25,893 participants (41.0%; 95% CI = 40.8%–41.6%) identifying as cisgender man. Thirty individuals indicated a binary transgender identity; 19 (0.03%; 95% CI = 0.02%–0.05%) and 11 (0.02%; 95% CI = 0.01%–0.03%) individuals identified as transgender woman and transgender man, respectively.

Eighty-one individuals (0.13%; 95% CI = 0.10%–0.16%) completed the fifth “Other than the above, namely …” response option to describe their sex assigned at birth and current gender identity. For these individuals the free-text field allowed for sharing experiences, which would not have been captured by pre-defined categorised answer options. Identified subgroups and exemplary answers are provided in Table 2, with 34 participants indicating a non-binary gender identity and 20 participants providing a binary answer that potentially could have fitted with the categorised answers, yet these participants chose to use the free-text field (e.g., “When I was born I was registered as a female, and I am a woman”). Such responses may indicate that participants did not fully understand the question or the response format. Reassigning these responses to the corresponding binary answer options, results in 36,848 cisgender women, 25,898 cisgender men, 21 transgender women and 11 transgender men. Finally, 13 participants provided an answer indicating annoyance or a joke, and 14 participants provided other responses, e.g., unclear answers or “n.a.” (Table 2).

**Table 2:** Identified subgroups in free-text answers regarding participants’ gender identity (N = 81).

Group	N (%)	Illustrative quote(s)
1) Non-binary identity	34 (42.0)	“Genderfluid, it differs per day” “I was registered as female when I was born, but I am non-binary (agender)”
2) Binary identity	20 (24.7)	“When I was born I was registered as a female, and I am a woman.” “I am a biological man.”
3) Annoyance or jokesters	13 (16.1)	“I will not cooperate in answering this nonsense.” “The fact I have to answer these questions everywhere is ridiculous and I won't do so, therefore not applicable.”
4) Unclear	9 (11.1)	“I am a human who is attracted to multiple sexes”
5) Not sure	2 (2.5)	“I don't really know.”
6) Blank/incomplete	2 (2.5)	“N.A.”
7) Intersex	1 (1.2)	“I was registered as male when I was born, but I was born with XXY”

**Fig. 1:**
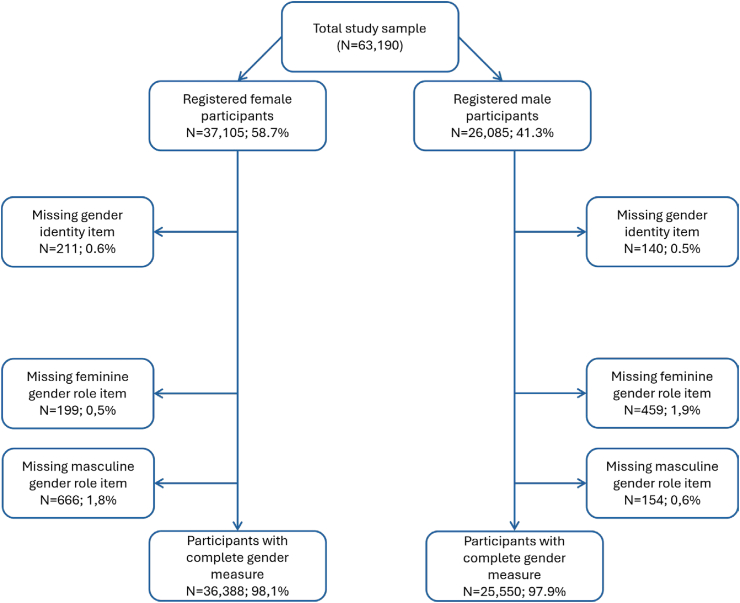
Missing data per gender identity and gender role measures, stratified by municipally registered sex.

There were 351 (0.6%) participants, of whom 211 (0.3%) were municipally registered as females, who did not complete the categorical gender identity item (Fig. 1).

### Dimensional assessment of gender role adherence by age and registered sex

**Fig. 2:**
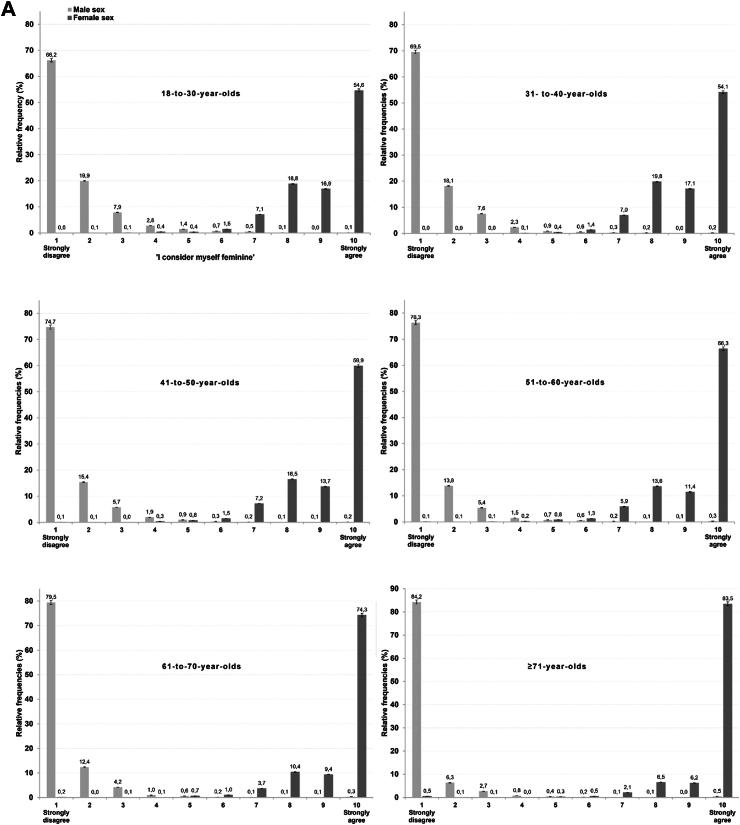

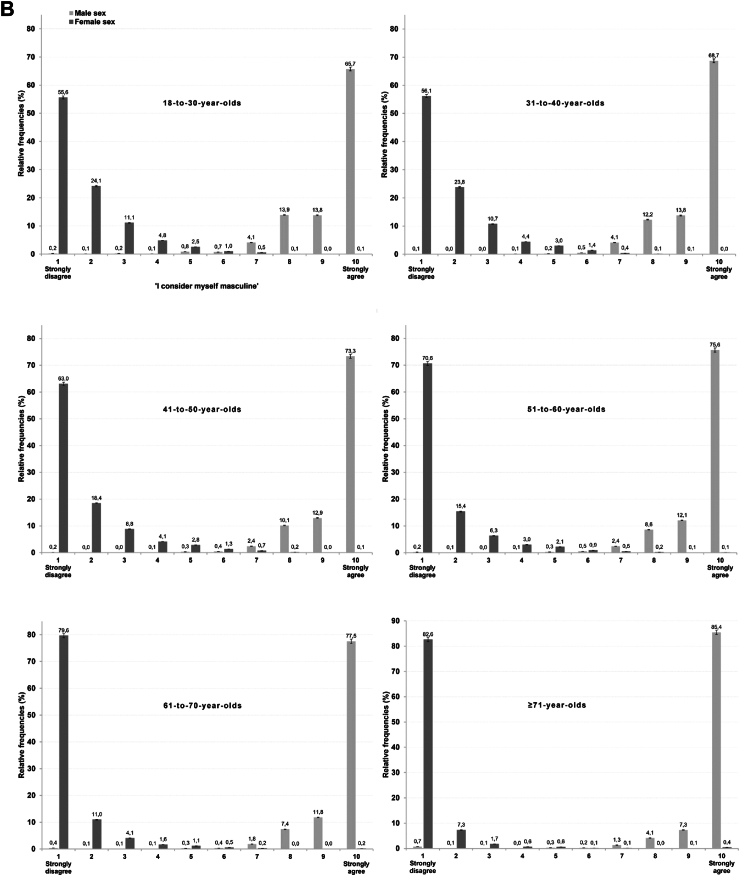
**(A)** Responses by all self-identified cisgender participants to the dimensional gender measure assessing femininity, stratified by municipally-registered sex and age category. Dark grey bars indicate participants registered as female, light grey bars indicate participants registered as male. **(B)** Responses by all self-identified cisgender participants to the dimensional gender measure assessing masculinity, stratified by registered sex and age category. Dark grey bars indicate participants registered as female, light grey bars indicate participants registered as male.

Fig. 2 shows the distribution of the proportion of self-identified cisgender participants indicating their (A) adherence to feminine and (B) masculine gender roles, stratified by registered sex and age category. Specifically, with regards to participants who were municipally registered as male, younger age groups scored lower on masculine gender role adherence than older age groups (*F*_(5,25925)_ = 35.5; *p* < 0.0001; η^2^ = 0.008 (95% CI = 0.006–0.010). A Bonferroni post-hoc test showed that the mean score of the youngest age group was 9.3 (SD = 1.2), while the oldest age group scored on average 9.7 (SD = 1.0; mean difference = −0.39, 95% CI = −0.51 to −0.28, *p* < 0.0001). Notably, 34.3% (95% CI = 33.8%–34.9%) of participants with a male sex registered by the municipality aged 18–to-30 years indicated that they did not fully adhere to masculine gender roles (i.e., ≤9 on the masculinity dimension). This proportion decreases to 14.6% (95% CI = 14.1%–15.0%) among participants aged 71 years or older.

A similar pattern regarding age groups was observed for self-reported adherence to feminine gender roles among participants who were municipally registered as female (*F*_(5,36900)_ = 137.2; *p* < 0.0001, η^2^ = 0.018 [95% CI = 0.016–0.021]). A Bonferroni post-hoc test showed that the mean score of the youngest age group was 9.1 (SD = 1.2), while the oldest age group scored on average 9.7 (SD = 1.0; mean difference = −0.55, 95% CI = −0.65 to −0.45, *p* < 0.0001). Of note, while 45.4% (95% CI = 44.9%–45.8%) of the 18–to-30-year-olds with a female sex registered by the municipality indicated that they did not consider themselves fully adhering to feminine gender roles (i.e., ≤9 on the femininity dimension), only 16.5% (95% CI = 16.1%–16.9%) of the participants aged 71 years or older did.

Irrespective of the age groups, participants who were municipally registered as males scored consistently and significantly higher on masculinity (M = 9.6; SD = 1.0) than participants who were municipally registered as female scored on femininity (M = 9.3; SD = 1.2; *t*_(60,459)_ = 27.7; *p* < 0.0001; Cohen's d = 0.218 (95% CI = 0.202–0.234)).

### Dimensional assessment of gender role adherence by gender identity and registered sex

Out of all cisgender participants 17 (0.05%; 95% CI = 0.03%–0.07%) of those who were municipally registered as female indicated predominantly adherence to masculine gender roles, and 11 (0.04%; 95% CI = 0.02%–0.08%) of the participants who were municipally registered as male indicated predominantly adherence to feminine gender roles. Furthermore, among all cisgender participants, 1242 participants who were (2.0%; 95% CI = 1.9%–2.1%) municipally registered as female and 421 participants who were (0.7%; 95% CI = 0.6%–0.7%) municipally registered as male were non-specifically highly gendered. In contrast, 87 (0.2%; 95% CI = 0.2%–0.3%) of the participants who were municipally registered as females and 84 (0.3%; 95% CI = 0.3%–0.4%) municipally registered as males were not highly gendered.

Among the 32 participants with a binary transgender identity six (18.8%; 95% CI = 5.2%–32.3%) transgender men indicated predominantly adherence to masculine gender roles, while 13 (40.6%; 95% CI = 23.6%–57.6%) transgender women indicated predominantly adherence to feminine gender roles. Out of 34 participants with a non-binary gender identity, 18 (52.9%; 95% CI = 36.2%–69.7%) were non-specifically highly gendered, while five (14.7%; 95% CI = 2.8%–26.6%) were not highly gendered. One participant with a non-binary gender identity indicated predominantly masculinity.

## Discussion

We quantified gender diversity in the Dutch general population cohort Lifelines. Out of 63,190 participants 32 indicated a binary transgender identity (21 transgender women and 11 transgender men after reassigning the free-text answers to respective binary categories) and 34 indicated a non-binary gender identity, resulting in 0.11% of the participants identifying as non-cisgender. In addition, among the cisgender participants, dimensional items on adherence to feminine and masculine gender roles revealed that younger participants and female participants showed relatively more diversity in gender than older participants and male participants. However, effect sizes were small. Our results show a missingness rate of 0.6%.

Using data from the UK Biobank (N∼487,600), a recent study found disagreement between participants’ chromosomal and self-reported sex in 0.04% of the cases.^22^ In that study, through linkage with medical records (e.g., diagnoses of Gender Dysphoria, or information on hormone therapy) the majority of sex discordances could be explained by intersex traits or a transgender identity.^22^ However, as more direct measures of gender were lacking, the number of participants with diversity in sex and/or gender was likely underestimated, explaining the difference with the proportion found in our study, i.e., 0.11% of participants indicating a non-cisgender identity.^22^ Our findings do align with a recent estimate of the Veterans Health Affair on non-cisgender identities.^27^ In contrast, international (census) studies in Europe and North America report higher estimates than found in our study. While 0.6% (N = 262,138) of respondents aged 16 years or older in the 2021 census study in the England and Wales indicated to identify differently than their sex registered at birth,^28^ 0.3% (N = 100,805) of respondents aged 15 years or older in the Canadian 2022 census release indicated a different gender identity than their sex assigned at birth.^7^ Statistics Netherlands provided a 1.0%-estimate of non-cisgender identities among the Dutch general population of 16 years and older,^29^ which aligns with earlier Dutch research among 8064 individuals, that indicates that 1.1% and 0.8% of participants with male or female sex assigned at birth, respectively, consider themselves a woman or man.^36^ A Swedish study among 50,157 respondents reported that 2.3% of participants feel like someone of a different sex.^37^

The measurements for diversity in gender identity differed across these (census) studies, in terms of including free-text answer options, cross-checks of current gender identity with self-reported sex or sex registered by the municipality, and phrasing of items related to gender identity. The age distribution of the study samples in these studies also differed from the current study, as the aforementioned studies include relatively more participants of a young age. Similarly, Lifelines includes a relatively high proportion of participants living in rural municipalities. This may partly explain the discrepancy in prevalence estimates between the current study and previous (census) studies. Notably, gender and its embodiment are highly dependent on culture, which may complicate international and temporal comparisons.

Previous studies suggested that non-cisgender identities are mainly prevalent among adolescents, compared to other age groups.^20^^,^^21^ This aligns with a recent study by Statistics Netherlands, that reports that of the 1.0% of the Dutch population that indicates a non-cisgender identity, 25.4% is aged between 15 and 24 years.^29^ Similarly, it is in line with US-based research showing that a non-cisgender identity is more common in young adult individuals (2.5% transgender identity and 0.8% other non-cisgender identity, N = 38,985 18–27-year-olds) compared to older individuals (0.1% transgender identity and 0.1% other non-cisgender identity, N = 114,558 68–77-year-olds) in the general population.^38^ We complement these findings in previous studies by reporting that the extent to which participants indicate to adhere to gender roles that are in line with their sex as registered by the municipality, increases with age. However, as all these studies used cross-sectional data on gender and gender diversity, it remains to be determined whether age or cohort effects best explain this phenomenon.

We found that participants with a female sex registered by the municipality reported greater diversity in gender than their male counterparts. This finding is corroborated by a previous study on sex-gender (in-)congruence among 736 Japanese university students; while about 15% of the males indicated complete sex-gender congruence, only 5% of the female participants did.^39^ This study combined elements of gender identity and gender roles to define sex-gender (in-)congruence. Also, despite the current study reporting lower estimates on non-cisgender identities, the results are in accordance with those of a previous Stockholm Public Health Cohort study, including over 50,000 participants from the general population.^37^ Assessing aspects of gender incongruence, they found 2.5% of female participants and 2.1% of male participants positively endorsed the item “I feel like someone of a different sex”. In addition, in line with our findings, the youngest group of 22–29-year-old participants showed higher rates (i.e., 4.0%) of gender incongruence than any of the older age groups (30–44 years: 2.5%; 45–66 years: 2.0%, 67+ years: 1.1%).^37^

We also found a high willingness among participants of a large-scale general population cohort to complete a self-report item on gender, with a missingness rate of 0.6%. Overall, our findings therefore challenge the notion that participants are hesitant or refuse to complete gender measures, as we find that the vast majority of participants provide an answer. This is in line with previous research conducted in the US (N = 645,720), that showed the percentage of missingness regarding items assessing gender identity increased from 0.55% among 18-to-27-year-olds to 1.9% among 68-to-77-year-olds.^38^ Similarly, a study assessing willingness to complete gender measures among 15,758 participants of the New Zealand general population reports a non-response rate of 0.5%.^40^

In our study, we found that only a small minority of individuals deliberately provided uninformative responses regarding gender, for example by expressing resistance, annoyance, or making jokes through the free-text field. The concept of ‘jokesters’, being individuals who respond mischievously to survey items regarding gender identity or sexual orientation in order to be funny, is well-known in research.^41^^,^^42^ As populations with a non-binary gender identity in large-scale general population cohort studies may be relatively small, the inclusion of jokesters herein may result in inflated differences in outcomes. To identify consistent jokesters, researchers typically use strategies such as a longitudinal follow-up to assess stability of the responses or triangulation of information from multiple informants (e.g., family members). Ultimately, these participants can be excluded from gender-focused analyses. While similar mechanisms as with jokesters may be at play in our study, merely a few free-text responses clearly show antipathy or political commentary towards gender identity measures. Again, this relatively small proportion of jokesters and participants displaying antipathy, provides an indication of participants' willingness to disclose information regarding their gender.

The findings of this study should be interpreted in the light of its limitations. First, the stratification of participants was based on sex registered by the municipality. However, in the Netherlands, individuals may change their registration in the municipal database, due to gender incongruent feelings. Therefore, for a small minority of people, sex may be reflective of their gender identity. The impact hereof in this study is not fully known, as it remains subject of debate what proportion of the Dutch population with gender incongruent feelings adapt their municipal registration.^43^ In addition, we report on two distinct approaches to measure individual's sex, namely the self-reported sex assigned at birth and sex as registered by the municipality, and transparently illustrate potential discrepancies between these. Second, even though the categorical gender item in Lifelines was an adaptation of the conventional two-step measure, it remained a binary measure that did not include an explicit option for non-binary sex and/or gender. Participants could indicate a non-binary sex or gender identity via the free-text field (i.e., “Other than the above mentioned options, namely …”). However, this may have inadvertently led to othering of gender diverse and non-binary individuals.^44^ Adding a categorised non-binary option for sex and/or gender would therefore be helpful, as would be rephrasing the free-text option into, for example, “Prefer to self-describe …”.^45^

This study also has several strengths. Foremostly, it leverages unique data from a large-scale general population cohort to explore detailed prevalences of gender identities, stratified by age and sex registered by the municipality. This study, therefore, fills a gap of knowledge regarding gender and its diversity in the general population across a wide age range, including older adults. Previously, such assessments were mostly limited to clinical and/or adolescent populations, or census studies.^20^^,^^21^^,^^38^ Another major strength of the study is that gender was assessed in a multi-dimensional way. We assessed participants' gender identity in relation to their self-reported birth-assigned sex, as well as their adherence to feminine and masculine gender roles. Our results show that providing participants with the option to indicate the latter on separate dimensions allows for a more nuanced understanding of the degree and the composition (i.e., predominantly masculine or feminine, neither, or both) of femininity and masculinity in the general population that moves beyond pre-defined gender categories. Last, it should be noted that the current gender measure in Lifelines largely aligns with five guiding principles formulated by the US National Academies of Sciences, Engineering, and Medicine in their 2022 report on inclusivity: (1) data collection practices that respect and reflect individual's identity; (2) precision in terminology; (3) respect for self-identification and autonomy, including an opt-out right; (4) parsimonious data collection practices and; (5) allowing for analysis that takes into account LGBTQI + communities' health.^46^^,^^47^

It has been argued that individuals do not fully separate gender and sex when reporting on their own lived experiences, but rather report hereon by using a mix of gender and sex.^8^^,^^9^ To move beyond the, according to some, false binary of sex as purely biological and gender as purely psychosocial, the integrative concept ‘gender/sex’ is used. In epidemiology, however, sex and gender are increasingly introduced as separate and conceptually different. Future research could assess to what extent the concept of gender/sex can be integrated and validated in large-scale general population cohort studies. Furthermore, we acknowledge that gender and its embodiment are strongly dependent on culture. Therefore, we recommend to develop gender measures, and potentially gender/sex measures, in collaboration with participant panels with lived experiences from the respective cultures. We strongly emphasise that the omission of items that assess dimensions of participants’ gender, or items that maintain the gender binary, is not recommended, as it may imply a denial of lived experiences for participants who identify beyond this binary.^48^ We argue for a recognition of diversity in aspects of gender, especially among the general public, and emphasise that this diversity should not be viewed as a pathology. Acknowledgement of diversity in gender contributes to its depathologization. At the same time, some individuals with non-cisgender identities may seek gender affirming care. General population cohort studies should include questions about the desire for such care, particularly among those identifying as non-cisgender. This would provide more accurate estimates and minimize selection bias. These insights could inform policy decisions, such as determining the need for more healthcare professionals specialized in gender-related care.

In conclusion, our study serves as an important first step toward enhancing our understanding of how sex and gender affect health and disease by reporting on their diversity. Future health research should integrate gender as a relevant individual difference variable, next to other socio-demographic variables, to examine its impact on health and disease across the lifespan.

## Contributors

SB developed the research idea, conceptualised the analyses, analysed the data, and provided input on the first draft of the manuscript. DK and LSK analysed the textual data, conceptualised its categorization, and provided input on all drafts and final version of the manuscript. AB developed the research idea, helped conceptualise the analyses, and led the writing of the manuscript. SB and AB had access to and verified the underlying study data.

## Data sharing statement

Lifelines data will not be shared publicly. Access to Lifelines data is organized according to a strict data access procedure. Researchers can apply to use Lifelines data via the Lifelines Research Office. Further information on Lifelines data, how to request this and the conditions of use can be found on Lifelines’ website (https://www.lifelines-biobank.com/).

## Declaration of interests

All authors declare no competing interest.

## Appendix A Supplementary data

Translated Abstract
