# Supplementary material for: Mapping diversity in gender identity and gender roles across sex and age in the Dutch general population: a large-scale cohort study
Source: eClinicalMedicine. 2025 Jul 28;86:103359. doi: 10.1016/j.eclinm.2025.103359 (PMC12399208; doi:10.1016/j.eclinm.2025.103359)
Supplement: Translated Abstract [file mmc1.docx]

***The following translations in Dutch were submitted by the authors and we reproduce them as supplied. They have not been peer reviewed. Our editorial processes have only been applied to the original abstract in English, which should serve as reference for this manuscript.***

**Dutch summary - Mapping diversity in gender identity and gender roles across sex and age in the Dutch general population: a large-scale cohort study**

Sarah M. Burke, Daniëlle B.A. Kroeze, S. Lucette Kiewiet, Aranka V. Ballering

**Diversiteit in genderidentiteit en gender rollen, gesplitst op geslacht en leeftijd, in kaart gebracht: een grootschalig cohort onderzoek**

**Achtergrond** Kennis over diversiteit in genderidentiteit en genderrollen in de algemene bevolking is beperkt. Deze studie heeft tot doel de prevalentieschattingen van genderidentiteit en de naleving van genderrollen onder de volwassen algemene bevolking te rapporteren, gestratificeerd naar leeftijd en bij de gemeente geregistreerd geslacht.

**Methoden** In de derde algemene meetronde van de prospectieve Nederlandse Lifelines Cohortstudie (2019–2023) werden geslacht en huidige ervaren genderidentiteit gemeten met een zelf gerapporteerd categorisch item, waarin deelnemers van ≥18 jaar hun bij geboorte toegewezen geslacht (man of vrouw) en ervaren genderidentiteit (man of vrouw) konden aangeven, of een openantwoordoptie konden kiezen. Twee afzonderlijke dimensionale items maten op een schaal van 1 tot en met 10 de mate van naleving van vrouwelijke en mannelijke genderrollen. Middels cross-sectionele data beschreven we de verdeling van genderidentiteiten en naleving van genderrollen, gestratificeerd naar leeftijd en geregistreerd geslacht. Verschillen werden getoetst met onafhankelijke t-toetsen en ANOVA.

**Resultaten** In totaal werden 63.190 deelnemers (gemiddelde leeftijd=55,4 jaar [SD=12,6]) geïncludeerd. De meerderheid van de deelnemers identificeerde zich als cisgender (36.835 [58,6%; 95%CI=58,2–58,9] cisgender vrouwen; 25.893 [41,2%; 95%CI=40,8–41,6] cisgender mannen). 66 (0,11%; 95% CI=0,08–0,13) deelnemers identificeerden zich als niet-cisgender. Bij cisgender deelnemers geregistreerd als man nam de naleving van mannelijke genderrollen toe met de leeftijd: 18–30-jarigen scoorden lager (M=9,3; SD=1,2) dan 71–97-jarigen (M=9,7; SD=1,0; F(5,25925)=35,5; p<0,0001; η²=0,008 [95%CI = 0,006–0,010]). Een vergelijkbaar patroon werd gevonden voor naleving van vrouwelijke genderrollen bij cisgender deelnemers geregistreerd als vrouw, waarbij 18–30-jarigen (M=9,1; SD=1,2) lager scoorden dan 71–97-jarigen (M=9,7; SD=1,0; F(5,36900)=137,2; p<0,0001; η²=0,018 [95%CI=0,016–0,021]). Cisgender deelnemers geregistreerd als man rapporteerden een sterkere naleving van mannelijke genderrollen (M=9,6; SD=1,0) dan cisgender deelnemers geregistreerd als vrouw van vrouwelijke genderrollen (M=9,3; SD=1,2; t(60,459)=27,7; p<0,0001; Cohen’s d=0,218 [95%CI=0,202–0,234]).

**Interpretatie** Hoewel de effectgroottes klein zijn, tonen jongeren en als vrouw geregistreerde individuen grotere diversiteit in genderrollen dan respectievelijk ouderen en deelnemers die als man zijn geregistreerd. Dit benadrukt dat diversiteit in naleving van genderrollen wijdverbreid is onder de Nederlandse volwassen bevolking. Een beperking van deze studie is de relatieve oververtegenwoordiging van oudere deelnemers, wat de generaliseerbaarheid naar jongere groepen beperkt. Klinisch en beleidsmatig wijzen deze bevindingen erop dat erkenning van genderroldiversiteit zorgverleners kan helpen interventies en behandeling aan het individu aan te passen. Gezien de kleine maar betekenisvolle effecten is vervolgonderzoek naar genderrollen en hun gezondheidseffecten in verschillende leeftijdsgroepen gewenst om de ontwikkeling van gendersensitief beleid te ondersteunen.
