# Supplementary material for: Mapping diversity in gender identity and gender roles across sex and age in the Dutch general population: a large-scale cohort study
Source: eClinicalMedicine. 2025 Jul 28;86:103359. doi: 10.1016/j.eclinm.2025.103359 (PMC12399208; doi:10.1016/j.eclinm.2025.103359)
Supplement: Appendix [file mmc2.pdf]

Appendix for: Mapping diversity in gender identity and gender roles across sex and age in the Dutch general population: a large-scale cohort study

Sarah M. Burke, Daniëlle B. A. Kroeze, S. Lucette Kiewiet, Aranka V. Ballering

Appendix 1. The self-reported gender measure included in the Dutch Lifelines Cohort study as of 2019.

Most people are born as either a man or a woman and they feel comfortable in a male or female body, respectively. However, this is not the case for everyone. Some people consider themselves a man, but were born in a female body or vice versa. Some people consider themselves neither a man nor a woman. Could you indicate which statement fits your experience best?

Could you indicate which statement fits your experience best?

() My sex assigned at birth was female and I currently consider myself a woman.

() My sex assigned at birth was male and I currently consider myself a man.

() My sex assigned at birth was female and I currently consider myself a man.

() My sex assigned at birth was male and I currently consider myself a woman.

() Other than the above-mentioned options, namely …

Some people, both men and women, consider themselves masculine, for example because they have characteristics or hobbies that most people consider masculine. On the other hand, both men and women may consider themselves feminine, because they have characteristics or hobbies that most people consider feminine. Some people consider themselves neither masculine nor feminine.

Could you indicate, on a scale ranging from 1 to 10 in which 1 equals *strongly disagree* and 10 equals *strongly agree*, to what extent you consider yourself feminine and masculine? Please complete both questions.

I consider myself feminine.

(1) Strongly disagree <<<<<<<<<< >>>>>>>>>> (10) Strongly agree

---

I consider myself masculine.

(1) Strongly disagree <<<<<<<<<< >>>>>>>>>> (10) Strongly agree

---

This table was modified from (Ballering et al., 2023)

Ballering, A. V, Burke, S. M., Maeckelberghe, E. L. M., & Rosmalen, J. G. M. (2023). How to ensure inclusivity in large-scale general population cohort studies? Lessons learned with regard to including and assessing sex, gender, and sexual orientation. *Archives of Sexual Behavior*, *52*, 2163–2172. https://doi.org/10.1007/s10508-023-02600-y
